# Effects of Cosmetic Formulations Containing Hydroxyacids on Sun-Exposed Skin: Current Applications and Future Developments

**DOI:** 10.1155/2012/710893

**Published:** 2012-05-20

**Authors:** Andrija Kornhauser, Sergio G. Coelho, Vincent J. Hearing

**Affiliations:** ^1^Office of Cosmetics and Colors, Center for Food Safety and Nutrition, US Food and Drug Administration, 4517 Pinecrest Heights Dr, Annandale, VA 22003, USA; ^2^Laboratory of Cell Biology, National Cancer Institute, National Institutes of Health, Bethesda, MD 20892, USA

## Abstract

This paper describes recent data on the effects of various skin formulations containing hydroxyacids (HAs) and related products on sun-exposed skin. The most frequently used classes of these products, such as **α**- and **β**-hydroxyacids, polyhydroxy acids, and bionic acids, are reviewed, and their application in cosmetic formulations is described. Special emphasis is devoted to the safety evaluation of these formulations, particularly on the effects of their prolonged use on sun-exposed skin. We also discuss the important contribution of cosmetic vehicles in these types of studies. Data on the effects of HAs on melanogenesis and tanning are also included. Up-to-date methods and techniques used in those explorations, as well as selected future developments in the cosmetic area, are presented.

## 1. Introduction

The cosmetic market is growing rapidly internationally and shows no sign of slowing in the foreseeable future. Within skin care products, antiaging and sun protection products are the main driving forces in this trend. Contemporary cosmetics contain a large number of active ingredients, such as botanicals, antioxidants, hormones, and hydroxyacids (HAs) to name just a few. In this paper, we focus on the role that HAs play in cosmetic/skin care products, their safety evaluations, and their effects on sun-exposed skin. This paper covers selected new developments in the field which appeared since our previous review of this topic, and it also includes selected topics not covered in that paper [1]. 

## 2. Presentation of HAs Structure and Classification

HAs have significantly influenced skin care since their introduction to dermatology about 40 yrs ago [2]. Since their inclusion in cosmetic formulations, they have been used to treat acne, ichthyosis, keratoses, psoriasis, photoaged skin and other disorders [3]. Following these developments, HAs have been gradually added into a variety of cosmetic products for daily use and over extended time periods [4]. At present, glycolic acid, lactic acid, and salicylic acid are the most frequently used HAs in cosmetics. One of the most cited beneficial effects of HAs is the reported improvement of photoaged skin. The driving force behind the increase in HAs use in cosmetic dermatology and skin care systems has been their antiaging effects [5]. Based on their structure and function, HAs can be classified as *α*-HAs, *β*-Has, and salicylic acid (SA) and its derivatives. The most common representative of an *α*-HA is glycolic acid, which was one of the first HAs to be incorporated into cosmetic formulations [4]. Another *α*-HA being used in various topical formulations is lactic acid (L-form).

Representatives of *β*-HAs are hydroxybutanoic acid, malic acid, and citric acid. Citric acid is presently widely used in various cosmetic formulations as an antioxidant [6]. SA and its derivatives are widely used in contemporary cosmetic formulations. In the cosmetic and dermatologic literature, SA is frequently described as a *β*-HA. As presented in a paper by Yu and van Scott, this classification is incorrect [7]. SA does not function as a *β*-HA according to its chemical structure or by its biological or physiological function [7].

**Figure 1:**
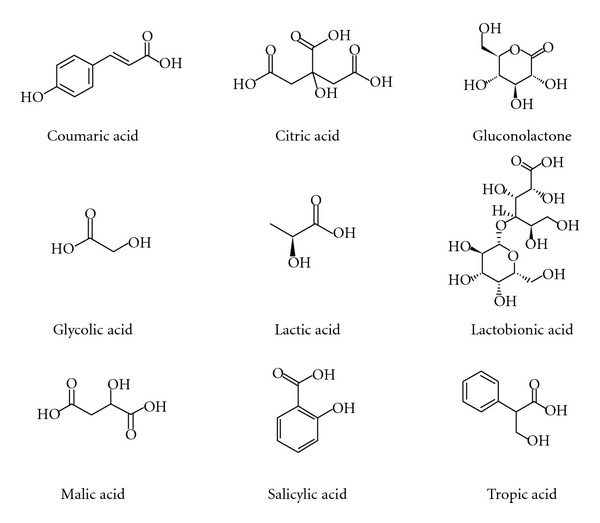
Structures of selected *α*-HAs, *β*-HAs, salicylic acid, and coumaric acid and two representatives of polyhydroxy acids, lactobionic acid, and gluconolactone.

Polyhydroxy acids (PHAs) and polyhydroxy bionic acids (PHBAs) constitute more recent arrivals into the cosmetic domain. They constitute a new generation of HAs with multiple skin benefits, making them very popular in cosmetic/skin care products. Structures of selected HAs are presented in Figure 1.

## 3. Effects of Formulations Containing HAs

Due to the rapid proliferation of HAs into the cosmetic world, safety evaluations of these products have became a crucial public health issue. As an important contribution to this problem, the Cosmetic Ingredient Review (CIR) Expert Panel assessed the available evidence and determined that *α*-HA ingredients are not reproductive or developmental toxins, mutagenic or carcinogenic, and skin sensitizers [8]. Furthermore, the CIR Panel recommended concentration (a maximum of 10%) and pH (at or above 3.5) in cosmetic formulations containing *α*-HAs. In addition, the CIR Panel recommended that HA-containing products should be formulated to avoid enhancing sun sensitivity and that consumers should be advised to use daily sun protection [8]. Several years later, the same CIR Panel evaluated products containing SA and salicylates and recommended that sensitivity to sunlight be taken into account when developing cosmetic formulations which was also the same recommendation provided in the initial assessment of *α*-HA-containing products [8, 9].

Knowledge of the involvement of any cosmetic/skin care product in the induction of photocarcinogenesis is still in its infancy. The largest and most comprehensive study so far, sponsored by the National Toxicology Program (NTP), reported on the photocarcinogenic potential of HAs [10]. The authors reported that glycolic acid did not modify the photocarcinogenesis induced by SSR, while SA (4%, cosmetic concentration) was photoprotective.

An important contribution to this field was recently published by Lu et al. [11]. They tested four commercially available moisturizing creams in their mouse model and found that, when applied topically to the mice, all the products had tumorigenic activity. This paper presents an important finding and deserves to be discussed here briefly. The concern about various effects of cosmetic creams on the skin is not entirely new. A number of published papers and clinical studies, including the above-mentioned NTP study, have suggested that there is a measurable influence of various applied cosmetic vehicles on cutaneous responses to UVR exposure. The protocol applied by Lu et al. differs significantly from previously reported approaches. Lu and his team applied the four selected creams to mice with a high risk of developing skin tumors without UVB exposure (termed high-risk mice) after the initial 3 months of exposure. In this case, the observed results cannot be explained by a vehicle effect on the optical properties of skin. Their paper, therefore, raises the question of whether these agents are more active compounds (tumor promoters). It was expected that such findings would result in immediate reactions. Two commentaries on Lu's paper were published in the same issue of the journal. The first commentary, by Staeb et al. [12], criticized the design of the experiments; the second commentary, by Ellefson [13], questioned the statistical approach. Both groups expressed some doubts about the validity of the findings of Lu et al. A third commentary, by Forbes [14], discussed the paper and provided a brief overview of the field. It is obvious that more studies of this kind are needed. The most important question emerging from this challenging laboratory data, however, is the relevance of these findings to humans. The cosmetic market is by no means static, and new products, including HAs and their derivatives, are steadily entering into the already large cosmetic repertory. A few examples will be highlighted here. Seo et al. [15] examined the effects of p-coumaric acid (PCA), a hydroxy derivative of cinnamic acid, on erythema and pigmentation in UV-exposed human skin. Twenty-one subjects, with the Fitzpatrick skin types III-IV, were included in the investigation. The authors report that topically applied PCA prevented UV-induced erythema and subsequent pigmentation in human skin. The authors speculated that PCA may suppress the induction of erythema by altering gene expression or by lowering the UV-induced inflammation. In addition, the authors suggested that PCA also acts as a potent tyrosinase inhibitor. In conclusion, Seo et al. stated that the product showed no apparent adverse reactions and, therefore, may be a useful active ingredient in cosmetic formulations. Im et al. [16] investigated the physicochemical properties of fatty ester derivatives of SA for UV protection. Octanoyl, nonanoyl, decanoyl, laurel, myristoyl, and palmitoyl oxysalicylate (C16SA) were investigated. The authors concluded that the C16SA was readily hydrolyzed to its parent compound in skin homogenates, suggesting that it might be converted to SA after topical administration. Furthermore, it showed the lowest permeation of SA in all types of skin and a lower accumulation and smaller uptake in the lipid phase as compared with the other derivatives examined. On the basis of the reported findings, they suggested that C16SA could be a potential candidate for UV protection. Another application of a SA derivative was recently reported by Merinville et al. [17]. They provided evidence that sodium salicylate (SS) obtained from the neutralization of 1% SA with sodium hydroxide can deliver satisfactory antiaging benefits, with significantly reduced skin irritation, which commonly occurs following application of SA to sensitive skin. They conducted three clinical studies, and by measuring biological, optical, and observational biomarkers they could demonstrate significant anti-aging effects of SS, which is especially suitable for subjects with sensitive skin.

Recently, Tasic-Kostov et al. [18] conducted a study to assess the safety and efficacy of lactobionic acid as compared to glycolic acid. Seventy-seven volunteers participated in the project, and skin color (erythema and melanin index), transepidermal water loss, electrical capacitance, and pH of the skin were measured. The authors confirmed that lactobionic acid resulted in improved skin benefits as compared with corresponding glycolic acid formulations, particularly with respect to skin irritation and barrier impairment.

## 4. Present Applications and Future Developments

Determining a person's sensitivity to sunlight is critical to understanding the UV effects of formulations with HAs. The MED is typically determined on each individual to assess their UV sensitivity in conjunction with knowledge of the Fitzpatrick skin type [19]. The Commission Internationale de L'éclairage (CIE) currently has a technical committee formed to address typical MEDs for individuals of all skin types, and their technical report will be forthcoming with recommendations and guidelines (http://div6.cie.co.at/?i_ca_id=609&pubid=318). The MED parameter allows one to compare biologically equivalent effects resulting from 1 MED to previous data in the literature. Typically, a series of exposures of increasing doses is used for MED determination as described in previous studies [20–24]. Subject phototype, site area size, geographical skin location, site-specific melanin content, and UV source vary by clinical study and present specific challenges when determining the MED. To allow for comparison across clinical studies, UV doses must be wavelength weighted using the CIE reference action spectrum for erythema [25]. Twenty-four hours after UV exposure, trained observers determine the MED by visual assessment using a scale where 1 MED is defined as pink erythema with at least one distinct border.

Obviously, this determination can be subjective and is highly dependent on a trained observer. There are a few limitations with assessing the MED visually especially when considering varying illumination conditions, environmental temperatures, and viewing angles during the visual assessment at different clinical sites. To address this issue, in the case of sunscreens there are some standard requirements regarding lighting conditions that have been set forth in the US FDA Sunscreen Monograph [26] that can also be applied to HA evaluation. In addition, increased melanin content presents a challenge when discriminating erythema, and this is an area where diffuse reflectance spectroscopy (DRS) excels over visual evaluation. Some pioneering work was performed over 2 decades ago that defined the use of CIE L*a*b* color space system variables and transformed them into a vector representation of skin pigmentation defined as the individual typology angle (ITA = [arctan (L*−50/b)] × 180/Pi) [27, 28]. However, this CIE L*a*b* system is inadequate to discriminate erythema masked by higher melanin content, and therefore measuring skin chromophores, such as melanin, oxyhemoglobin and deoxyhemoglobin, by DRS is highly desirable for defining the MED noninvasively. Several studies have been published on the use of DRS, which prove that it can be applied as an adjunct to aid in the prediction and measurement of MEDs for HA UV effects [29–31].

In the past decade, gene array technologies have changed the way we evaluate skin biology responses by allowing us insight into the global genome profile at particular moments in time and under specific environmental conditions. Recently through the use of microarrays the different spectral responses from repetitive UVA and/or UVB exposure of human skin were characterized [32]. Further, acute doses with full-spectrum solar-simulated radiation and solar-simulated UVA with or without SPF 15 sunscreen treatment were also assessed [33]. However, there are virtually no published microarray studies evaluating HA formulations with relation to the effects of UV on skin, but there have been some interesting studies that have evaluated skin responses with or without UV when pretreated with skin lightening or irritating compounds [34, 35]. This is an area that has great potential to become the method of choice for safety screening of cosmetic preparations. Approaches to evaluate global gene expression patterns correlated to cosmetic outcomes may also provide insights into the profiles of responders and nonresponders to formulations containing HAs.

## 5. Effects of HAs on Pigmentation

HAs have also been shown to have significant effects on skin pigmentation. *α*-HAs, such as glycolic acid and lactic acid, have been shown to be effective in treating various types of hyperpigmentary lesions, such as photoaging [36, 37], melasma [38–40], solar lentigines [41, 42], and postinflammatory hyperpigmentation [43]. The proposed mechanism underlying those effects is thought to involve epidermal remodeling and accelerated desquamation, which results in a more rapid and efficient dispersion of existing pigment. Usuki et al. reported an in vitro study [44] which showed that glycolic acid and lactic acid suppress melanin synthesis by inhibiting tyrosinase activity, the critical enzyme involved in melanin synthesis, in human and in mouse melanoma cells. Both the transcription and translation of tyrosinase were decreased significantly, which resulted in the reduced enzyme function but with no significant effect on cell growth. Based on that in vitro analysis, increased epidermal turnover and inhibition of melanin formation were surmised to be the effects of glycolic acid and lactic acid treatment. The potential effects of HAs on skin pigmentation resulting from UV exposure (i.e., tanning) have been less well characterized. UV-induced effects of glycolic acid on skin pigmentation were investigated in both Asian and Caucasian subjects who received UVA and UVB irradiations on both sides of their lower backs, and on the contralateral extensor forearms [45]. Treatments were applied once daily for 1 week and then twice daily for 7 weeks; as a control, a placebo gel was applied on the opposite sides [45]. Areas pretreated with glycolic acid had increased UVB-induced tanning on the forearm and the lower back in both races compared to the untreated control areas. In contrast, only Asian subjects showed increases in tanning on the exterior forearms. The use of PHAs on photoaged skin in several skin phototypes has also been recently investigated [46]; the results show that, in addition to several beneficial effects on skin physiology, PHAs elicit a significant skin lightening, although the mechanism by which that occurs has not yet been elucidated. Glycolic acid has recently been assessed with respect to its efficacy when used in combination with skin peels elicited by nonablative lasers, intense pulsed light, and trichloroacetic acid. That study showed that glycolic acid had synergistic effects when used with those other treatments to improve skin parameters, including lightening of the skin and smoothing uneven pigmentation [47]. The majority of those clinical studies were performed with subjects having skin types I–III, and to address similar issues of the effects of HAs on subjects with darker skin types investigations using darker skin types (IV–VI) are needed.

## 6. Conclusions

HAs and related products, such as PHAs and PHBAs, are now standard ingredients in cosmetics and skin care products, as well as in a number of dermatologic formulations. They can be applied in various types of vehicles, integrated into liposomes, or in a variety of nanoparticle formulations. We still need more information about the safety of the long-term use of these products. This is particularly evident for the effects of HAs on sun-exposed skin. Topically applied HAs can interact with many basic molecular processes occurring in mammalian skin (summarized in Table  1 of  [1]), such as cell proliferation, cytokine production, apoptosis, antioxidants potencies, and others. Recent investigations clearly demonstrated that the effects of topically applied HAs are not limited exclusively to their often cited keratolytic properties. New methodologies, some of them included in this paper, are needed and are already being applied in designing contemporary cosmetic products, as well as in assessing their safety.

## Abbreviations

BA: Aldobionic acid
DRS: Diffuse reflectance spectroscopy
HA: Hydroxyacid
*α*HA: *α*-Hydroxyacid
*β*HA: *β*-Hydroxyacid
MED: Minimal erythema dose
PHA: Polyhydroxy acid
PHBA: Polyhydroxy bionic acid
ROS: Reactive oxygen species
SSR: Solar-simulated radiation
UV: Ultraviolet.
